# A New Subtype of Hepatitis C Virus Genotype 3: Analysis of Available Evidence

**DOI:** 10.5812/hepatmon.13380

**Published:** 2013-08-17

**Authors:** Faraz Salehi Moghadam, Seyed Reza Mohebbi, Seyed Masoud Hosseini, Behzad Damavand, Mohammad Reza Zali

**Affiliations:** 1 Gastroenterology and Liver Diseases Research Center, Shahid Beheshti University of Medical Sciences, Tehran, IR Iran; 2 Department of Microbiology, Faculty of Biological Sciences, Shahid Beheshti University, Tehran, IR Iran

**Keywords:** Hepatitis C, Genotype, Phylogeny, Iran

## Abstract

**Background:**

Hepatitis C virus (HCV) is one of the leading causes of chronic liver disease. Seven genotypes and more than 80 subtypes have been identified for HCV so far. To date, 10 subtypes (3a to 3i; and 3k) of HCV genotype 3 have been identified. In 2006, two HCV isolates were reported from Iran that belonged to a new subtype of genotype 3. However, considering the consensus proposal for HCV genotype nomenclature, the available sequences of the new subtype did not correspond to the regions that are required to be analyzed prior to subtype assignment. During a study on the molecular epidemiology of HCV in Iran, an HCV isolate (FSM165) which seemed to belong to a new subtype of genotype 3 was obtained from a patient residing in Tehran, Iran.

**Objectives:**

The aim of this study was to assess the relatedness of isolate FM165 together with several sequences retrieved from the database to the new HCV-3 subtype reported from Iran in 2006.

**Materials and Methods:**

Various parts of the genome including the core/E1 region and two segments of the NS5B region were amplified and sequenced for isolate FSM165. Furthermore, using the Basic Local Alignment Search Tool (BLAST), the HCV database was searched for sequences that had a high level of similarity with sequences of FSM165 isolate and such sequences were retrieved from the database. To investigate the relatedness of isolate FSM165 and also the retrieved sequences to a new HCV-3 subtype reported previously, phylogenetic analyses were performed using the Kimura two-parameter model and the neighbor joining method.

**Results:**

Phylogenetic analysis of the partial NS5B region demonstrated the relatedness of isolate FSM165 to the new subtype reported from Iran in 2006. Moreover, some core/E1 and NS5B sequences that had a high level of similarity with FSM165 isolate were found through searching the HCV database. These sequences were previously either misclassified or could not be accurately classified. Phylogenetic analyses showed that all of the described sequences belonged to the new subtype of HCV genotype 3.

**Conclusions:**

Data suggests that the new subtype has a vast geographical distribution in Iran. The core/E1 and the NS5B sequences described in this paper can be used as references for the new HCV-3 subtype in future studies.

## 1. Background

Hepatitis C virus (HCV) is one of the main causes of chronic liver disease and has infected approximately 170 million people worldwide (1). HCV is a member of the Flaviviridae family which consists of enveloped viruses with single stranded positive sense RNA. The viral genome is about 9.6 kb in length and consists of two untranslated regions at 5’ and 3’ ends and a single open reading frame (ORF) encoding a polyprotein precursor of about 3000 amino acids (2). Based on the nucleotide sequence of the genome, six major genotypes and more than 80 subtypes have been identified thus far. Furthermore, a complete genomic sequence of a candidate for the seventh genotype has been deposited in the database (3). Based on the whole genome nucleotide sequence, genetic distances among various HCV genotypes are about 31-33%, compared with 20-25% among subtypes (4).

Various HCV genotypes have different geographical distribution patterns. Genotypes 1, 2 and 3 are distributed throughout the world (5-7). In the Middle East, genotype 4 is predominant in Arab countries whereas 1b and 3 are predominant in Turkey and Pakistan, respectively (8, 9). Several previous studies have shown that 1a is the most frequent subtype in Iran, followed by subtypes 3a and 1b (10-13).

To date, 10 subtypes of genotype 3 have been identified (3a to 3i; and 3k). Only three of these subtypes (a, b and k) have been confirmed so far and the remaining seven subtypes have been provisionally assigned. Moreover, a complete genomic sequence of a genotype-3 isolate with a new subtype was reported recently (14).

In 2006, Amini et al. reported two Iranian isolates as candidates for subtype 3l (15). They reported that based on the phylogenetic analyses of 5’UTR, core and NS5B regions, the two isolates belonged to genotype 3 but they formed a cluster separated from known subtypes of genotype 3. Considering the consensus proposal for HCV genotype nomenclature (4), however, the analyzed sequences did not correspond to the regions that are required to be analyzed prior to subtype assignment.

During a study on the molecular epidemiology of HCV in Iran, we identified an isolate (FSM165) that seemed to belong to a new subtype of genotype 3. In this study, the relatedness of the mentioned isolate to the new subtype reported from Iran in 2006 is demonstrated. Moreover, we found more sequences of the new subtype by searching the HCV database. These sequences were previously either misclassified or could not be accurately classified.

## 2. Objectives

The aim of this study was to phylogenetically analyze and report the isolates that seemed to belong to a new subtype of genotype 3.

## 3. Materials and Methods

### 3.1. Amplification and Sequencing of Isolate FSM165

Viral RNA was obtained from a plasma sample using QIAamp Viral RNA mini kit (QIAGEN, Hilden, Germany). Complementary DNA was synthesized using Random Hexamer primers and RevertAid Reverse Transcriptase enzyme (Thermo Fisher Scientific Inc.). A partial segment of the NS5B region corresponding to positions 8616-9113 of H77 reference sequence (AF009606) was amplified using polymerase chain reaction (PCR) with primers HCV8619M13F and HCV9090M13R (16). The thermal conditions were 5 min at 95 °C followed by 35 cycles of 1 min at 94 °C, 1 min at 60°C and 45s at 72 °C. Final elongation step was performed for 10 min at 72 °C.

Another segment of the NS5B region corresponding to positions 8260–8639 of H77 reference sequence was amplified using semi-nested PCR. Primers hep-101 and hep-120 were used for the first-round PCR and primers hep-101 and hep-105 were used for the second-round PCR. Thermal conditions were described previously (17, 18).

The core/E1 region corresponding to positions 843-1316 of H77 reference sequence was amplified using primers 493S_H77 (493) and 987R_H77 (987) for the first-round PCR and primers 502S_H77 (502) and 975R_H77 (975) for the second-round PCR. Thermal conditions were described elsewhere (19).

Table 1 shows nucleotide sequences of the primers used in this study. PCR products were sequenced bi-directionally and sequences were edited using BioEdit version 7.0.5.3 (20). 

**Table 1.** Nucleotide Sequences and Positions of the Primers Used in This Study

Region	Primer Name	Primer Sequence	5' Position	Reference
**NS5B**	HCV8619M13F	5'- TTCACGGAGGCTATGACYAG -3'	8616	(16)
	HCV9090M13R	5'- TGCCCGATGTCTCCAAGCTCGTA -3'	9113	(16)
	hep-101	5'- ATACCCGCTGCTTTGACTC -3'	8260	(18)
	hep-120	5'- TGCGCGACBGABACRTTKGAGGA -3'	8722	(17)
	hep-105	5'- ATACCTAGTCATAGCCTCCGTGA -3'	8639	(18)
**Core/E1**	493S_H77 (493)	5'- GCAACAGGGAACCTTCCTGGTTGCTC -3'	834	(19)
	987R_H77 (987)	5'- CGTAGGGGACCAGTTCATCATCAT -3'	1328	(19)
	502S_H77 (502)	5'- AACCTTCCTGGTTGCTCTTTCTCTAT -3'	843	(19)
	975R_H77 (975)	5'- GTTCATCATCATATCCCATGCCAT -3'	1316	(19)

### 3.2. Searching the HCV Data Base

Search for possible similar sequences to the amplified segments was performed through Basic Local Alignment Search Tool (BLAST) and such sequences were retrieved from NCBI GenBank. Furthermore, reference sequences for all of the seven HCV genotypes and all subtypes of genotype 3 were retrieved from the Los Alamos HCV sequence database (21), regardless of their types of assignments (confirmed, provisional or unassigned).

### 3.3. Phylogenetic Analyses

Sequences retrieved from the data base together with the corresponding sequences obtained from the patient were aligned using ClustalX version 2.0.12 (22). Phylogenetic trees of the NS5B and the core/E1 regions were constructed using the Kimura two-parameter algorithm (23) with the neighbor-joining method. Genetic distances were also calculated using the Kimura two-parameter model. MEGA software version 5 was used for the analyses (24).

## 4. Results

**Figure 1.**
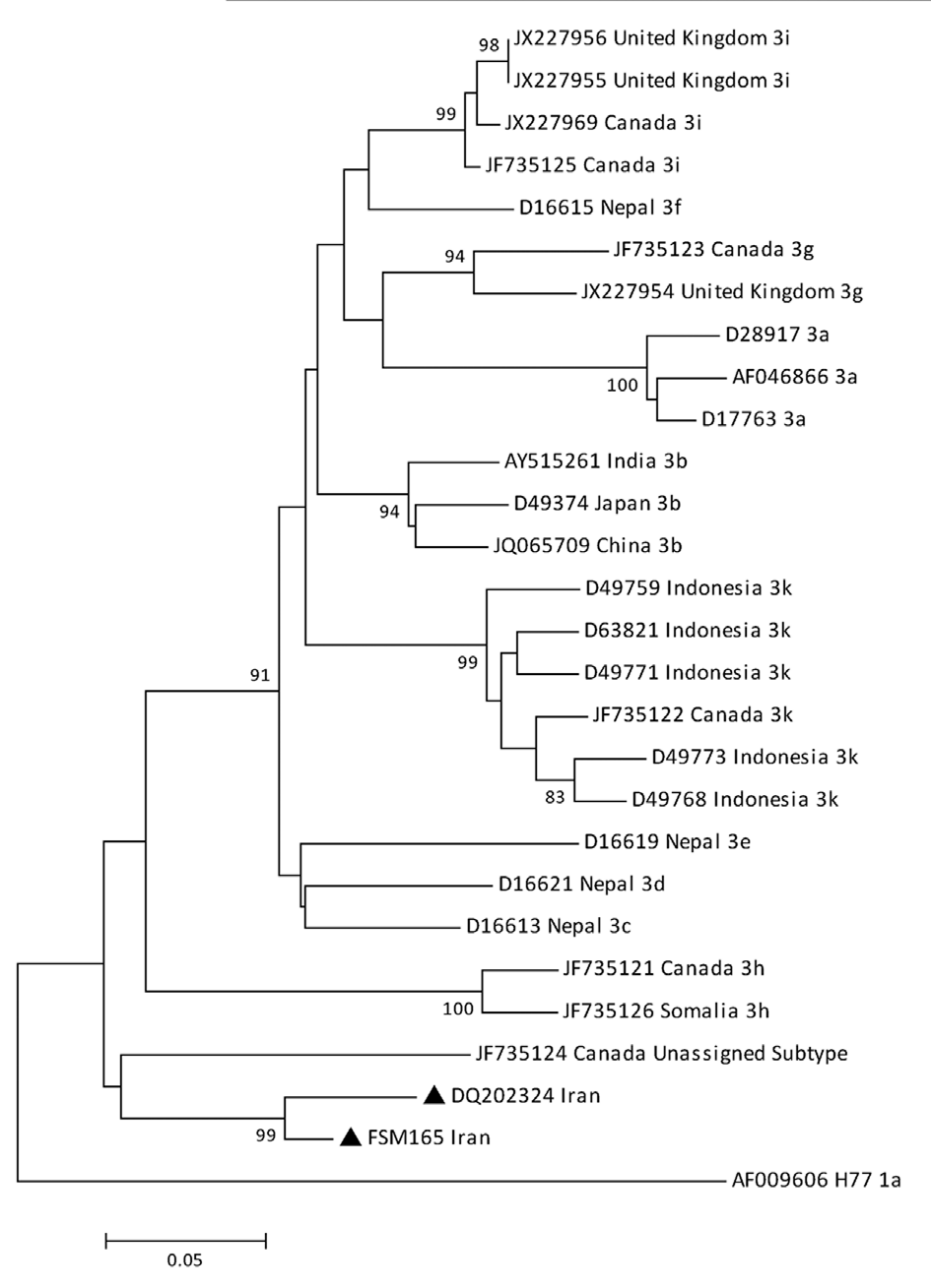
Phylogenetic Tree Constructed Based on the Partial Segment of the NS5B Region Corresponding to Positions 8761-9005 of H77 Reference Sequence Using the Neighbor Joining Method The HCV isolates with the new subtype (indicated by black triangles) were compared with various subtypes of genotype 3. Reference sequences are shown by their GenBank accession numbers and country of isolation. Numbers at the nodes show the percentages of bootstrap values (1000 replicates). H77 reference sequence was used as an out-group

Core/E1 region and two different segments of the NS5B region were successfully amplified and sequenced for FSM165 isolate. To investigate the relatedness of our newly identified isolate, FSM165, to the new HCV-3 subtype previously reported from Iran, we compared their NS5B sequences corresponding to positions 8615-9080 of H77 reference sequence (AF009606). In the phylogenetic tree constructed based on the mentioned region, the two isolates formed a separated cluster from other HCV-3 subtypes with a bootstrap value of 99% (Figure 1). Furthermore, the mean genetic distance between the NS5B sequences of these two isolates was calculated to be 5.5%. Table 2 shows the genetic distances between these two isolates and other HCV-3 subtypes. These results revealed that the two isolates belonged to the same subtype of genotype 3. 

**Table 2.** The Mean Genetic Distances Between the Two Isolates With a New Subtype and Various Subtypes of Genotype 3 Based on the Nucleotide Sequence of the NS5B Region (Positions 8761-9005)

Subtype/Isolate	Distance From, %	
	FSM165	DQ202324^b^
**FSM165**		5.5
**3a**	24.7	27.5
**3b**	25.3	25.3
**3c**	19.6	21.8
**3d**	20.1	24.1
**3e**	21.7	24.7
**3f**	20.1	21.8
**3g**	20.1	21.6
**3h**	19.6	20.8
**3i**	18.5	22.4
**3k**	23.3	24.8
**QC115^a^**	17.9	19.6

^a^Belongs to a new but not-yet-assigned subtype of genotype 3

^b^No isolate name was assigned to this sequence

Apart from the sequences reported by Amini et al. in 2006 (15), no other corresponding sequence of the new subtype has been deposited in the database. When belonging of the FSM165 isolate to the new subtype was revealed, we amplified and sequenced two other segments of the genome including the core/E1 (positions: 843-1316) and the NS5B (positions: 8260–8639) regions. According to the HCV genotype nomenclature ( 4 ), sequences of these two regions are required to assess whether an isolate belongs to a new subtype. BLAST analyses were performed to compare the similarity of the sequenced regions of FSM165 isolate with other sequences in the database. One core/E1 sequence (isolate C4; JN129986) and two NS5B sequences (isolate N4; JN129985 and isolate 934; AY654000) were found to have a similarity of more than 90% with the corresponding sequences of isolate FSM165. Interestingly, all of these sequences were related to HCV isolates obtained from Iranian patients. Two of the retrieved sequences (JN129985 and JN129986) were obtained from an HIV/HCV co-infected patient in Tehran. These two sequences were deposited in the database with two different isolate names (C4 and N4). However, according to a preliminary report, both of these sequences were related to one isolate (25). This isolate is called "C4/N4" in the present article. In the database, both of the mentioned sequences were described as genotype 3, without designating any specific subtype. The third sequence (AY654000) was related to isolate 934 which was obtained from a hemodialysis patient in Tehran and reported in 2004 (13). Due to some unintentional mistakes, however, this isolate was classified as genotype 5. Table 3 shows available information for all of the discussed sequences. 

**Table 3.** Available Data on the Isolates With the New Subtype of HCV Genotype 3

Isolate	Year	Patient				Available sequences			Reference
		Place	Age	Sex	Known risk Factor	Genomic Region	Nucleotide Position^a^	Accession Number	
**?^b^**	2006	Khouzestan	57	male	nd^c^	Core	342 – 704	DQ065830	(15)
									
**?^b^**	2006	Lorestan	25	male	IV drug abuse	5'UTR	315-142^d^	DQ202322	(15)
						Core	342 - 661	DQ202323	
						NS5B	8615 - 9080	DQ202324	
									
**934**	2004	Tehran	nd	nd	hemodialysis	NS5B	8266 - 8561	AY654000	(13)
									
**C4/N4**	2010	Tehran	41	nd	nd	Core/E1	842 - 1317	JN129986	(25)
						NS5B	8253 - 8651	JN129985	(25)
									
**FSM165**	2011	Tehran	39	male	cupping, tattooing	Core/E1	842 - 1317	KF218587	-
					periodontal procedure	NS5B	8263 - 8636	KC285335	-
						NS5B	8761 - 9005	KF218588	-

^a^Nucleotide positions are based on H77 reference sequence (AF009606)

^b^No name was assigned to this isolate

^c^nd, no data was available

^d^Deposited sequence was related to the reverse strand

**Figure 2.**
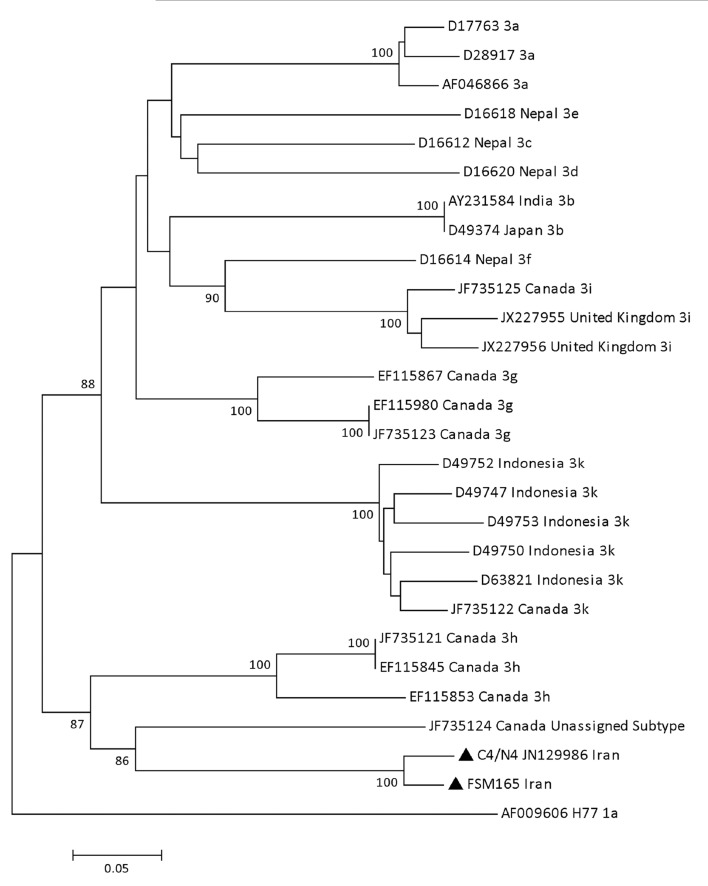
Phylogenetic Tree Constructed Based on the core/E1 Region Using the Neighbor Joining Method The HCV isolates with the new subtype (indicated by black triangles) were compared with various subtypes of genotype 3. Reference sequences are shown by their GenBank accession numbers and country of isolation. Numbers at the nodes show the percentages of bootstrap values (1000 replicates). H77 reference sequence was used as an out-group.

In the phylogenetic tree constructed based on the core/E1 region, isolates FSM165 and C4/N4 formed a cluster (bootstrap value: 100%) in proximity of an isolate recently reported from Canada (Figure 2). The subtype of the Canadian isolate (QC115) has not yet been assigned at the time of the preparation of this article. The mean genetic distance between the core/E1 sequences of the isolates FSM165 and C4/N4 was calculated to be 5%. Table 4 shows the mean intra-genotypic distances between the discussed isolates and all subtypes of genotype 3, regarding the nucleotide sequence of the core/E1 region. 

**Table 4.** The Mean Genetic Distances Between FSM165 and C4/N4 Isolates and Various Subtypes of Genotype 3, Based on the Nucleotide Sequence of the core/E1 Region

Subtype/Isolate	Distance From, %	
	FSM165	C4/N4
**C4/N4**	5	0
**3a**	44.1	45.6
**3b**	46.1	46.1
**3c**	47.4	46.9
**3d**	44	41.3
**3e**	44.3	45.3
**3f**	44.8	43.9
**3g**	41.7	42.8
**3h**	36.3	37.9
**3i**	47.6	45.1
**3k**	45.5	47.4
**QC115^a^**	33.8	33.9

^a^Belongs to a new but not-yet-assigned subtype of genotype 3

**Figure 3.**
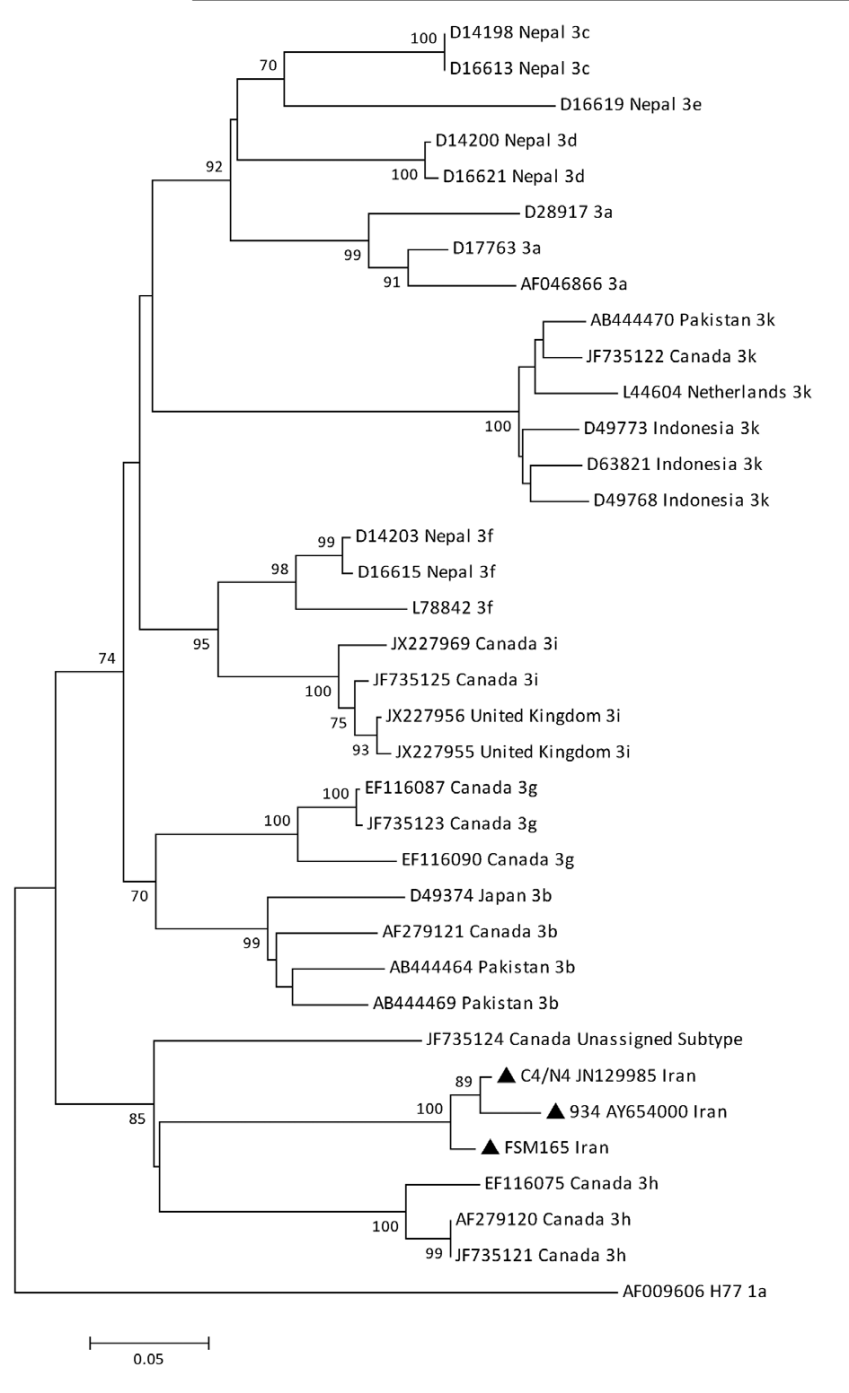
Phylogenetic Tree Constructed Based on the Partial Segment of the NS5B Region Corresponding to Positions 8282-8561 of H77 Reference Sequence Using the Neighbor Joining Method The HCV isolates with the new subtype (indicated by black triangles) were compared with various subtypes of genotype 3. Reference sequences are shown by their GenBank accession numbers and country of isolation. Numbers at the nodes show the percentages of bootstrap values (1000 replicates). H77 reference sequence was used as an out-group.

As Figure 3 shows, in the phylogenetic tree of the NS5B region, isolates FSM165, C4/N4 and 934 formed a cluster (bootstrap value: 100%) between HCV subtype 3h and isolate QC115 with an unassigned subtype. Table 5 shows the mean intra-genotypic distances based on the partial nucleotide sequence of the NS5B region. The genetic distance between isolates FSM165 and C4/N4 was 2.99%, between isolates FSM165 and 934 was 4.5% and between isolates C4/N4 and 934 was 2.99%. In comparison with other subtypes, these three isolates were genetically more close to isolate QC115 with an unassigned subtype (mean genetic distance: 23.6 – 28%) and HCV subtype 3h (mean genetic distance: 26.1 – 27.9%). 

**Table 5.** The mean genetic distances between isolates FSM165, C4/N4 and 934 and various subtypes of genotype 3, based on the nucleotide sequence of the NS5B region (positions 8282-8561)

Subtype/Isolate	Distance From, %		
	FSM165	C4/N4	934
**C4/N4**	2.9		
**934**	4.5	2.9	
**3a**	36.1	37.8	39.8
**3b**	33	33	34.5
**3c**	36.9	36.8	40.8
**3d**	31.7	33.4	35.3
**3e**	35	34.9	34.9
**3f**	34.6	34.8	36.9
**3g**	30.2	31.9	33.5
**3h**	26.5	26.1	27.9
**3i**	32.6	33.1	36.8
**3k**	35.4	35.9	37.7
**QC115^a^**	23.6	25.8	28

^a^Belongs to a new but not-yet-assigned subtype of genotype 3

The above results showed that isolates FSM165, C4/N4 and 934 should be classified as a new subtype of HCV genotype 3.

## 5. Discussion

According to the consensus proposal for HCV genotype nomenclature published in 2005 (4), for provisional subtype assignment two criteria should be met: (1) at least three examples of infection with the new subtype should be described, otherwise the subtype remains unassigned; (2) sequences of both the core/E1 and the NS5B regions should be analyzed. The core/E1 sequence should correspond to at least 90% of nucleotides of positions 869 - 1292 in the H77 reference sequence (AF009606). The NS5B sequence should correspond to at least 90% of nucleotides of positions 8276 – 8615 in the H77 reference sequence.

In 2006, Amini et al. reported two Iranian HCV isolates that belonged to a new subtype of genotype 3 (15).

According to their report, for both isolates, core regions (DQ202323 and DQ065830) were analyzed. For the second isolate, 5’UTR (DQ202322) and NS5B (DQ202324) regions were also analyzed. Regarding the analyzed segments of the genome, these two isolates were genetically more close to subtypes 3h and 3k, in comparison with other subtypes of genotype 3. The genetic distances of the new isolates from subtypes 3h and 3k, however, were further from where they could be classified as any of these two subtypes. Eventually, this report suggested that the new isolates could be provisionally assigned as subtype 3l, although the positions of the reported sequences did not correspond to the positions that are required to be analyzed prior to subtype assignment.

Since 2006, no other report has been published about this new subtype. This might be mainly due to the fact that in the only published study (15), reported sequences were from parts of the HCV genome that did not correspond to the core/E1 and the NS5B regions usually used for HCV genotyping. Consequently, although some other sequences were deposited in the HCV database, the relatedness of these sequences to the new subtype remained undiscovered.

In this study, we sequenced different parts of the genome of an Iranian HCV isolate (FSM165), which seemed to belong to a new subtype of genotype 3. We assessed the relatedness of this isolate to the new subtype reported in 2006 and found that isolate FSM165 was related to the new HCV subtype. Furthermore, we found other sequences in the HCV database and demonstrated that they also belonged to this new subtype.

According to our results, the discussed isolates should be classified as a new subtype of HCV genotype 3. Considering the 2005 consensus proposal (4), however, these isolates do not have the criterion of availability of both the core/E1 and the NS5B sequences obtained from at least three unrelated infected individuals. It seems that there is a lack of complete agreement on assigning a name to this new subtype. In the Los Alamos HCV database, the new subtype has been provisionally assigned as 3l whereas in the European HCV database no such subtype has been considered among provisional subtypes of genotype 3. Very recently, complete genomic sequence of a new but yet-unassigned subtype of genotype 3 was reported from Canada (14). Assigning a subtype to the new Canadian isolate in the near future would clarify the issue of whether the new Iranian subtype could be provisionally assigned as subtype 3l or it should be assigned with the next available letter once all of the sequences required by the 2005 consensus proposal are available. Needless to say, full-genome sequence characterization would be of utmost significance and lead to confirmation of this new subtype.

Currently, epidemiological data on the new HCV-3 subtype is lacking. The new subtype has been isolated from infected individuals residing in three provinces of Iran: Tehran (Central North), Lorestan (Central West) and Khouzestan (South West). Considering these regions, it seems that the new subtype has a vast geographical distribution in Iran. However, isolation of this subtype from other countries has not been reported so far. Thus, it seems that this HCV-3 subtype is endemic in Iran. The frequency of this subtype among Iranian HCV-infected individuals is unknown. According to the results of this study, only 5 isolates with the new subtype have been identified so far. This is not necessarily indicative of the low prevalence of the new subtype among Iranian patients because there are only a few studies, that used a phylogenetic approach to investigate HCV epidemiology in Iran. Therefore, more phylogenetic studies are necessary to determine the frequency of this new subtype.

In conclusion, phylogenetic analyses revealed the relatedness of an HCV isolate obtained from an Iranian patient in this study together with several HCV nucleotide sequences from the data base to a new subtype of genotype 3. It seems that the new subtype is endemic in Iran and it has been circulating among Iranian HCV-infected individuals for several years. Moreover, evidence shows that the new subtype has a vast geographical distribution in Iran. The core/E1 and the NS5B sequences described in this paper can be used as references for the new HCV subtype in future studies and pave the way for provisional assignment of it.
